# Selection of the fusion and fixation range in the intervertebral surgery to correct thoracolumbar and lumbar tuberculosis: a retrospective clinical study

**DOI:** 10.1186/s12891-021-04335-0

**Published:** 2021-05-21

**Authors:** Zongqiang Yang, Changhao Liu, Ningkui Niu, Jing Tang, Jiandang Shi, Zili Wang, Huiqiang Ding

**Affiliations:** 1 Department of Spine Surgery, General Hospital of Ningxia Medical University, 804 Shengli Street, Xingqing District, Yinchuan, 750004 People’s Republic of China; 2 Department of Orthopedics, Zhangye People’s Hospital Affiliated to Hexi University, 67 Huancheng West Road, Zhangye, 734000 People’s Republic of China

**Keywords:** Disease intervertebral surgery, Non-disease intervertebral surgery, Thoracolumbar tuberculosis, Lumbar tuberculosis

## Abstract

**Background:**

To compare the diseased verses the non-diseased intervertebral surgery used in the treatment of thoracolumbar and lumbar spinal tuberculosis and to explore the best choice of fusion of fixation range.

**Methods:**

Two hundred twenty-one patients with thoracolumbar and lumbar tuberculosis were categorized into two groups. One hundred eighteen patients underwent the diseased intervertebral surgery (lesion vertebral pedicle fixation, Group A) and 103 patients underwent the non-diseased intervertebral surgery (1 or 2 vertebral fixation above and below the affected vertebra, group B). Spinal tuberculosis diagnosis was confirmed in both groups of patients before lesion removal, bone graft fusion, and internal fixation. Clinical data and efficacy of the two surgical methods were then evaluated.

**Results:**

The mean follow-up duration for both procedures was 65 months (50–68 months range). There were no significant differences in laboratory examinations, VAS scores, and the Cobb angle correction rate and the angle loss. However, significant differences existed in the operation time, blood loss, serosanguineous drainage volume, and blood transfusion requirement between the two groups. The diseased intervertebral surgery group performed significantly better than the non-diseased intervertebral surgery group in all of these areas. In both cases, the bone graft fused completely with the normal bone by the last follow-up, occuring at 50–86 months post surgery.

**Conclusion:**

The diseased intervertebral surgery is a safe and feasible option for the treatment of thoracolumbar and lumbar tuberculosis. It effectively restores the physiological curvature of the spine and reduces the degeneration of adjacent vertebral bodies in the spinal column.

## Background

**Fig. 1:**
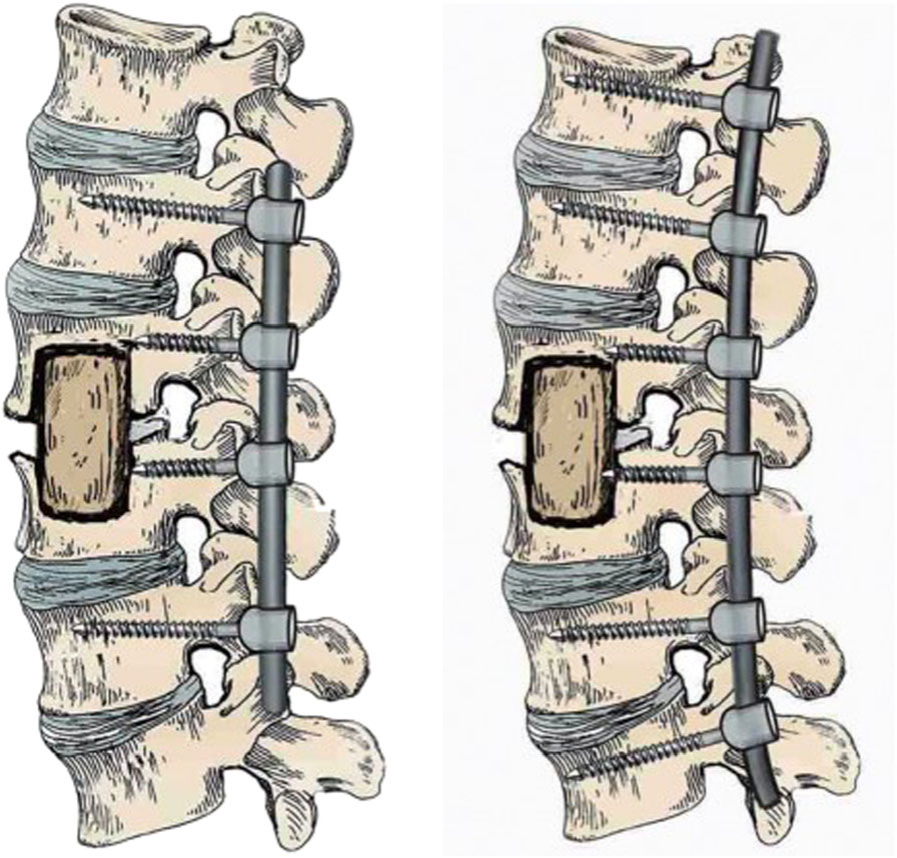
short-and long-segment fixation

Spinal tuberculosis is a chronic non-specific infectious disease caused by *Mycobacterium tuberculosis*, of which thoracic tuberculosis (48.03%) and lumbar tuberculosis (42.36%) are the most common [1]. Anti-tuberculosis drugs can be effectively used to cure spinal tuberculosis [2, 3]. However, surgery is required for patients with severe spinal deformity, vertebral instability, cold abscess formation, spinal cord nerve injury, and paraplegia. In such cases, surgery can be used to significantly improve its cure rate and reduce recurrence and related complications [4, 5]. A typical surgery protocol includes removal of the spinal tuberculosis lesions followed by autologous bone defect repair and lastly, internal fixation to restore the biomechanical stability of the spine [6, 7]. However, there are no clear guidelines or literature on the fixation procedure used to stabilize the spine. The vertebral body from the thoracic 10 to sacral 1 is a completely isolated bone structure, and reconstruction of the spine after the removal of tuberculosis lesions in this segment is challenging. As a result, the clinical scope of internal fixation here is much debated. The conventional fixation method for spinal reconstruction, otherwise known as the non-diseased intervertebral surgery, is further divided into two categories: short-and long-segment fixation (Fig. 1). The short-segment fixation is achieved by fixing a normal motor unit above and below the diseased vertebrae respectively. The long-segment fixation, on the other hand, is characterized by fixing two or more normal motion units above and below the diseased vertebra respectively. During both of the non-diseased intervertebral fixation procedures, part or all of the posterolateral normal intervertebral space fuse with the diseased intervertebral space [8, 9]. Although the fixation and fusion methods mentioned above may meet the biomechanical requirements of the spine, they often sacrifice the normal motor units of the spine, leading to the degeneration of adjacent segments and the occurrence of chronic lower back pain [10]. It is, therefore, crucial to consider reducing the fixation range while maintaining the mechanical strength of the reconstructed spine.

**Fig. 2:**
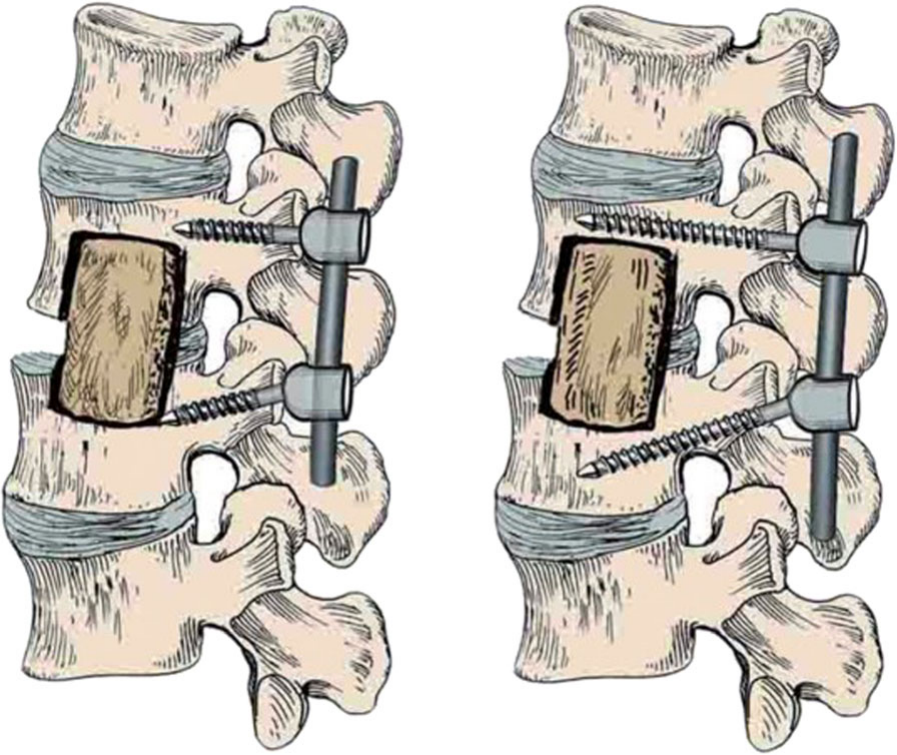
the diseased intervertebral

The single-segment fixation, otherwise known as the diseased intervertebral (Fig. 2 and has been successfully applied to the treatment of thoracic and lumbar spine fractures [11, 12]. In this study, a continuous single-segment surgery method for multi-segment spinal tuberculosis was adopted. The procedure involved complete removal of the lesion, bone graft fusion, and internal fixation of the instrument performed only in the motor unit involved in the lesion, and without any involvement of the adjacent normal motor units. As a result, unlike non-diseased intervertebral fixation, the adjacent segments remained intact and unharmed by the fixation procedure.

In this paper, we retrospectively evaluated the clinical efficacy of the diseased intervertebral surgery verses the non-diseased intervertebral surgery in the treatment of thoracolumbar and lumbar tuberculosis at the Department of Spine Surgery in the General Hospital of Ningxia Medical University in Ningxia, China. Our goal is to provide a future reference for the effective selection of fusion and fixation range in the treatment of spinal tuberculosis.

## Methods

### Inclusion and exclusion criteria

Inclusion criteria: 1) disease is diagnosed based on clinical manifestations, laboratory and imaging examinations; 2) little to no pedicle damage; 3) absence of severe osteoporosis; 4) no other health complications (for example, severe liver and kidney dysfunction) and able to tolerate surgery; and 5) indications for spinal tuberculosis debridement.

Exclusion criteria: 1) those with severe kyphosis in the active stage of spine tuberculosis and those unable to tolerate surgery; 2) those whose spine tuberculosis was in stationary phase or fully cured but required osteotomy orthopedics; and 3) cases with incomplete data and those missing follow-up.

### General information

**Fig. 3:**
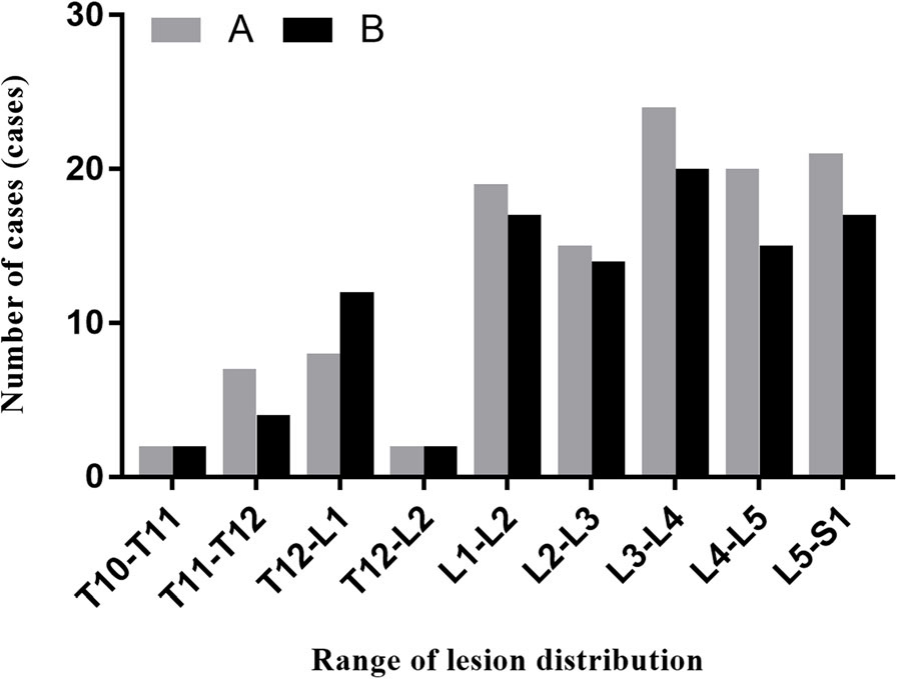
Distribution of the spinal tubercuiosis lesions in 221 patients

We performed a retrospective analysis of 221 cases of thoracolumbar and lumbar tuberculosis admitted to our Department of Spine Surgery from January 2012 to June2018. Out of the 221 cases, 118 were subjected to the diseased intervertebral surgery and 103 received the non-diseased intervertebral surgery. The 118 cases in the diseased intervertebral surgery group (pedicle screw fixation group, group A), exhibited abscess formation: 40 cases of psoas major abscess, 17 cases of paravertebral abscess, 4 cases of lumbar triangle, and 4 cases of popliteal; combined deformities: 6 cases of kyphosis and 4 cases of scoliosis. The 103 cases from among the non-diseased intervertebral surgery group (1 or 2 vertebral fixation groups in the upper and lower vertebral bodies, group B) also exhibited abscess formation: 26 cases of psoas abscess, 19 cases of paraspinal abscess, 3 cases of lumbar triangle abscess, and 5 cases of popliteal abscess; combined deformities: 13 cases of kyphosis and 3 cases of scoliosis, and two groups Frank classification of neurological function as shown in Table 6. The distribution of the lesions in both groups is shown in Fig. 3, and the general preoperative information of the two groups is shown in Table 1.

**Table 1:** Comparison of the general clinical data between Groups A and B

Item	Group A(118cases)	Group B(103 cases)	Test value(*t/x*^*2*^)	*P-*value
Age	38.84 ± 15.41	40.66 ± 15.61	*t* = 0.393	*P =* 0.695
Male/female	56/62	47/56	*x*^*2*^ = 0.489	*P* = 0.446
Course of disease (months)	16.46 ± 16.79	17.21 ± 20.28	*t* = 0.303	*P* = 0.762
ESR (mm/h)	37.49 ± 23.62	37.58 ± 22.74	*t* = 0.303	*P* = 0.976
CRP(mg/L)	24.72 ± 26.25	26.22 ± 23.13	*t* = 0.446	*P* = 0.656
Cobb angel(°)	17.03 ± 18.95	15.91 ± 12.80	*t* = 0.508	*P* = 0.612
VAS score (points)	6.15 ± 1.74	5.72 ± 1.62	*t* = 1.91	*P* = 0.057

### Preoperative preparations

Patients in both groups were bedridden before surgery, and given isoniazid (0.3 g/d), rifampicin (0.45 g/d), pyrazinamide (0.75 g/d), and ethambutol (0.75 g/d) for anti-tuberculosis treatment for more than 2 to 3 weeks; blood sedimentation rate was decreased to less than 30 mm/h or more (The normal range for ESR is male 0–15 mm/h, female is 0–20 mm/h), and tuberculosis control was achieved. Cough, fever, night sweats, fatigue, and other symptoms of systemic tuberculosis poisoning were relieved. During the perioperative period, hypoproteinemia was corrected, nutrition supplemented, hemoglobin maintained above 100 g/L (The normal range for Hb is 130-175 g/L), and normal liver and kidney functions confirmed before continuing with the regular anti-tuberculosis drugs. In other words, all complications of system-related diseases were addressed and spinal surgery was performed only under conditions of zero obvious surgical contraindications.

### Surgery group and method

According to the scope of the surgery, patients were grouped into either the diseased intervertebral surgery (Group A) or the non-diseased intervertebral surgery (Group B). All patients underwent general anesthesia with posterior instrument internal fixation (diseased intervertebral fixation or non-disease intervertebral fixation), primary or staged anterior lesion removal, and intervertebral support bone graft fusion.

#### Posterior internal fixation instruments

**Fig. 4:**
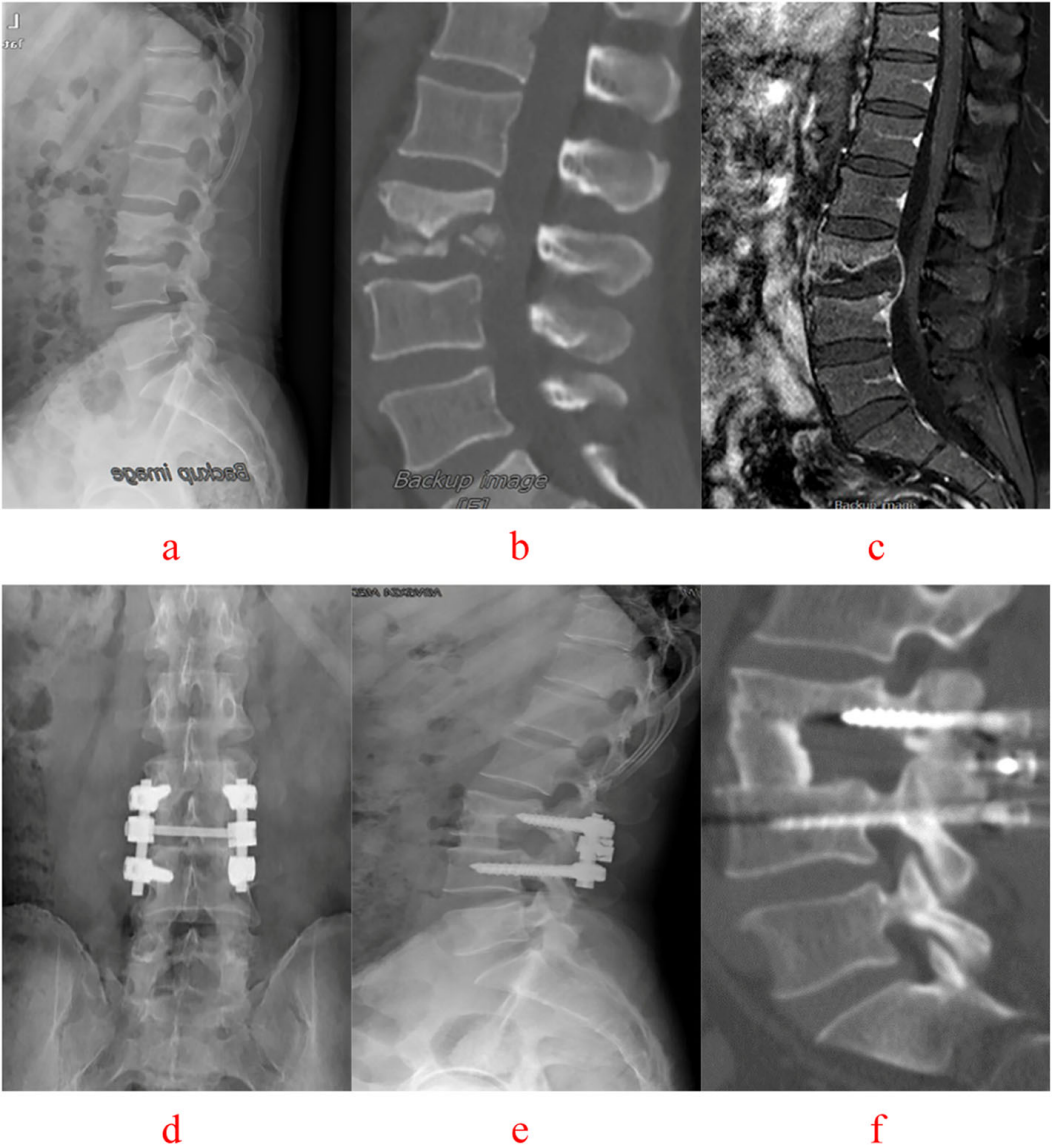
A 47-year-old male with L3–4 vertebral tuberculosis underwent intervertebral surgery by combined posterior-anterior approaches. The preoperative images (**a** X-ray, **b** CT reconstruction and **c** MRI) showeddestruction of the L3–4 intervertebral space and nerve compression. Postoperative X-ray (**d**, **e**), 64 months after surgery, showed that intervertebral fixation was excellent, and the L3 vertebrae are fixed with short pedicle screws and CT reconstruction (**f**) illustrated that L3–4 vertebral tuberculosis was completely cured, bone graft fusion and no obvious correction angle loss with good fixation position

**Fig. 5:**
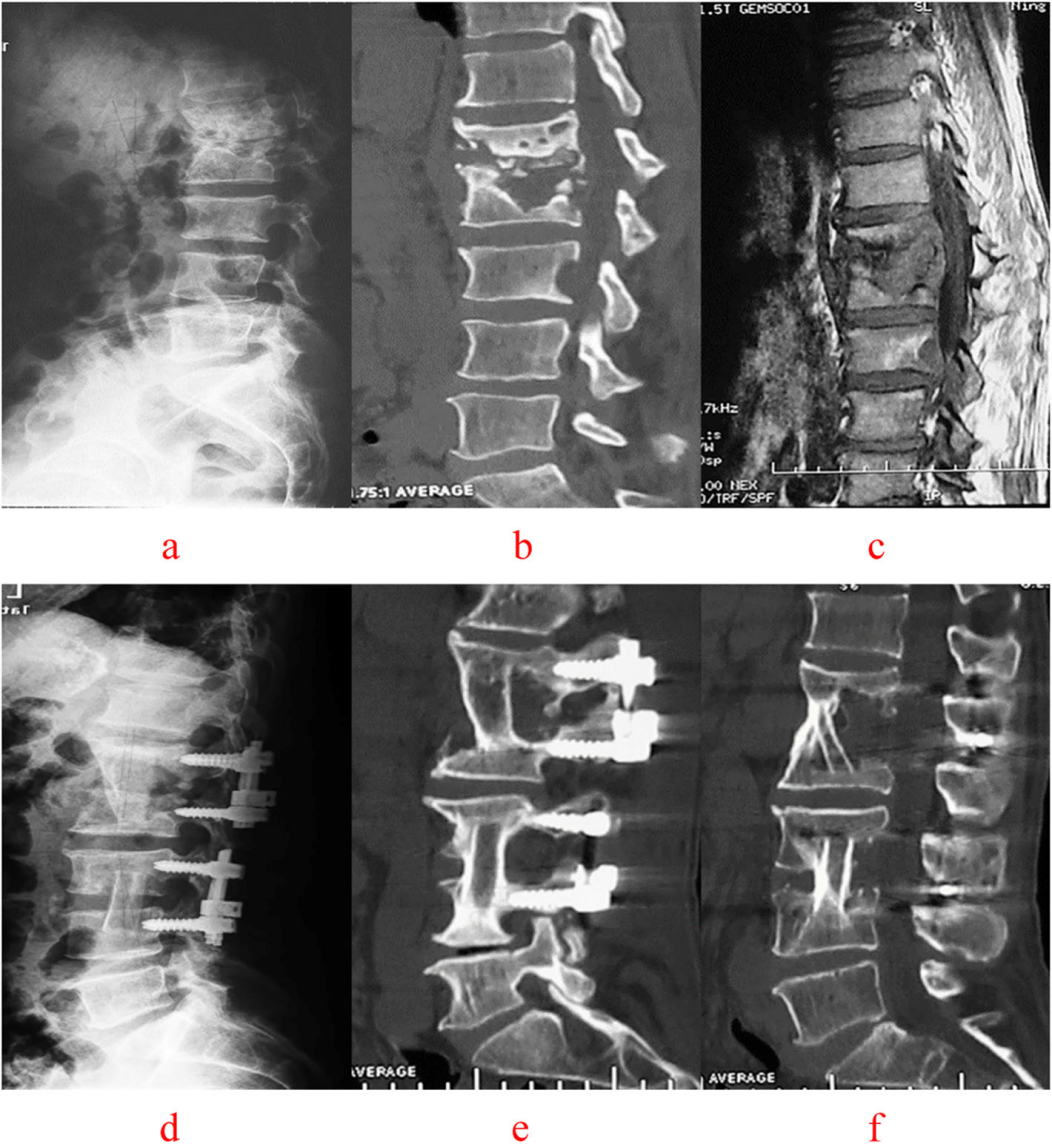
A 55-year-old male with L1–2 and L3–4 vertebral tuberculosis underwent intervertebral surgery by combined posterior-anterior approaches. The preoperative images (a X-ray, b CT reconstruction and c MRI) showed destruction of the L1-L2, L3–4 intervertebral space and spinal cord compression. During the follow-up, at 1 month(d) and 36 months(e) after surgery, X-ray presented the strut bone is located firmly between the affected vertebrae, intervertebral fixation is excellent, and the L1–2 and L3–4 vertebrae are fixed with short pedicle screws and There was no degeneration of adjacent segments and no loss of physiological curvature of the lumbar spine. 72 months after surgery CT reconstruction (f) illustrated that L1–2 and L3–4 vertebral tuberculosis was completely cured, bone graft fusion and no obvious correction angle loss with good fixation position

**Fig. 6:**
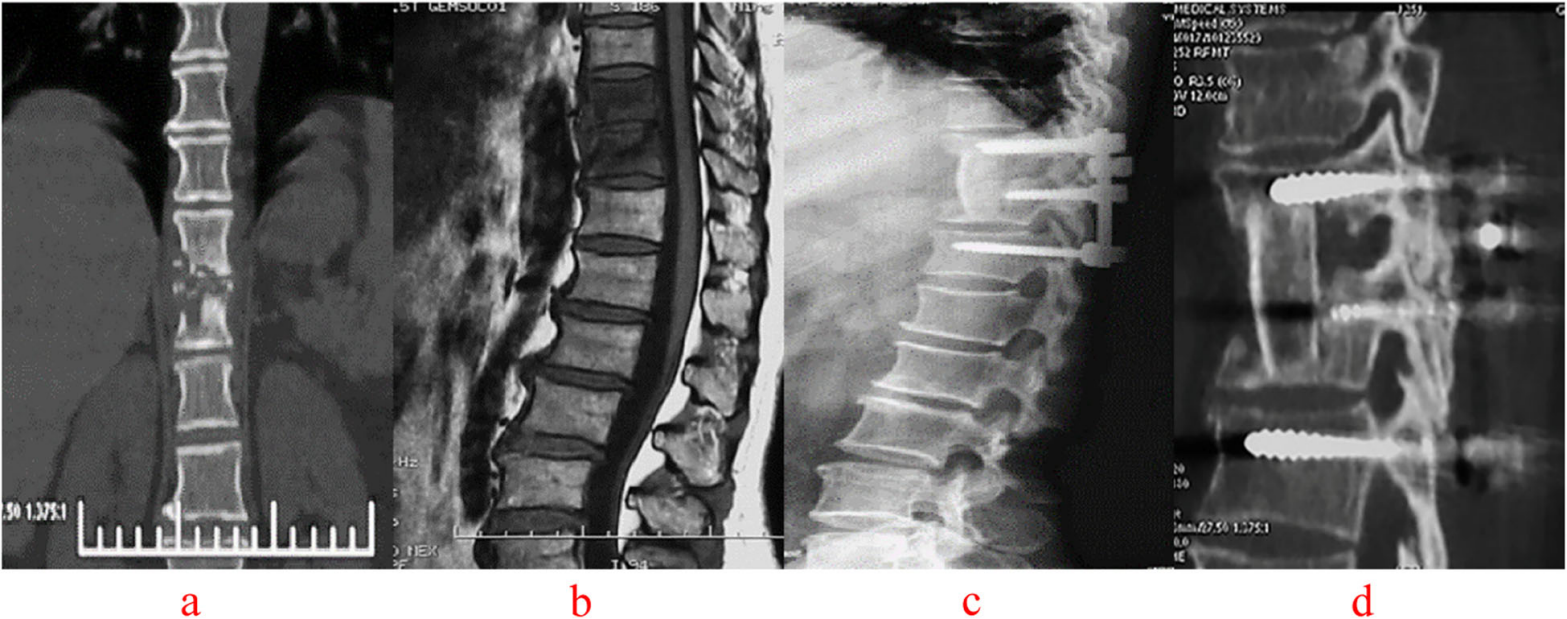
A 32-year-old female with T10–11 vertebral tuberculosis underwent non-diseased intervertebral surgery by combined posterior-anterior approaches. The preoperative images (**a** CT reconstruction and **b** MRI) showed destruction of the T10–11 intervertebral space and spinal cord compression. The postoperative images (**c** X-ray and **d** CT reconstruction) 42 months after surgery illustrated that T10–11 vertebral tuberculosis was completely cured, bone graft fusion and no obvious correction angle loss with good fixation position

**Fig. 7:**
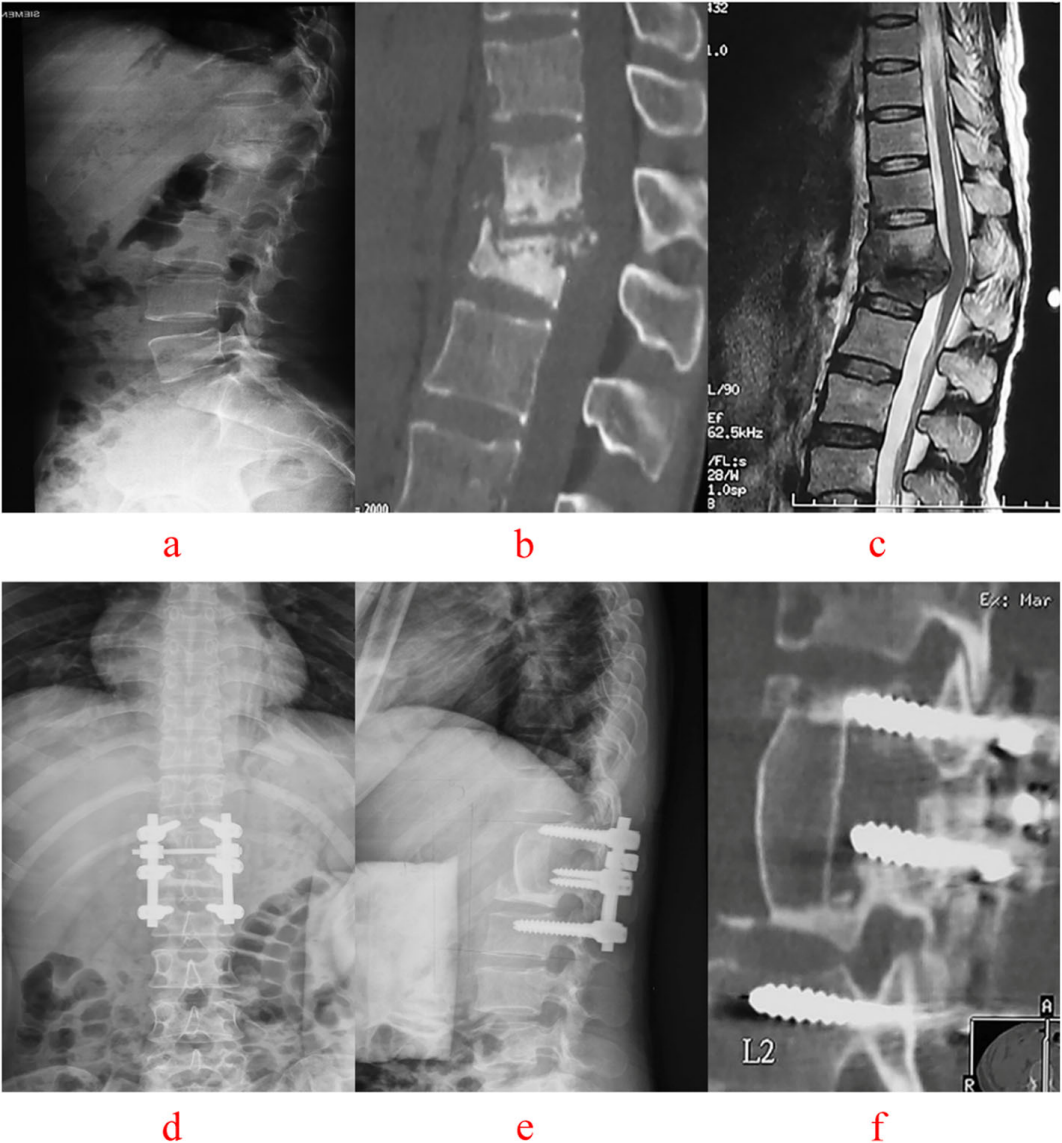
A 47-year-old male with T12-L1 vertebral tuberculosis under wentnon-diseased intervertebral surgery by combined posterior-anterior approaches. The preoperative images (**a** X-ray, **b** CT reconstruction and **c** MRI) showed destruction of the T12-L1 interverte bral space and spinal cord compression. Postoperative X-ray (**d, e**), 64 months after surgery, showed that intervertebral fixation was excellent, and the L1 vertebrae are fixed with short pedicle screws and CT reconstruction (**f**) illustrated that L3–4 vertebral tuberculosis was completely cured, bone graft fusion and no obvious correction angle loss with good fixation position

C-arm fluoroscopy was used to locate the vertebral lesion(s). A posterior midline incision was used to expose the diseased vertebra (Groups A, Figs.4, 5), along with the upper and lower normal vertebrae (Group B, Figs.6, 7). The flesh was removed layer by layer and the lateral process was exposed on both sides. Next, both Group A and B received transpedicular instrument fixation kyphosis correction and diseased intervertebral lamina decortications followed by screw fixation and interlaminar spinous process vertebral joint fusion.

#### Anterior lesion removal and intervertebral bone grafting

Various anterior surgical approaches were used in different areas of the spinal tuberculosis. The thoracolumbar joint or extra peritoneal approach was used in the thoracolumbar segment; the lateral renal incision was used in the upper lumbar spine; and the lower lumbar and sacral vertebrae were treated by the supine inverted “eight” incision through the peritoneum. The lesion size was determined by the preoperative imaging examination, and the lesion exposure and resection range were determined by the degree of spinal cord compression and the distribution range of intraspinal, or paraspinal abscess. The anterior lesion removal approach was selected according to the severity of the vertebral damage and the size of abscess. The abscess was exposed layer by layer. First, a thick needle was used to detect the accurate position of the abscess, then the site was expanded and the pus was expelled with a suction device, the abscess cavity was opened and the abscess moss and case-like substances were scraped off. To find the bone fistula hole, the diseased vertebral body was established along the orifice of the bone. Next, the vessels of the vertebral segment were ligated, and the damaged bone of the diseased disc and vertebral body were fully exposed and removed [13, 14].

Any dead bones, abscesses, granulomas, necrotic intervertebral discs or other tissues that protruded from the spinal canal, were removed to relieve compression to the spinal cord, dural sac, and nerve roots. On occasion when the spinal canal was not involved, the posterior edge and the back of the vertebral body longitudinal ligament was not opened to prevent *Mycobacterium tuberculosis* and necrotic materials from entering the spinal canal and contaminating it. In short, bone knife or scraping instruments were used for the removal of diseased bone from the edge of the lesion to the periphery of the lesion until the location of the section under direct vision resembled gravel-like bone. The diseased bone, on the other hand, was identified as bone that failed to harden, was full of dead space, had the consistency of cheese, and contained granulation tissue. After the lesions were completely removed, the wound was repeatedly washed with normal saline. On occasion when the vertebral endplate bone could be preserved during the resection of the vertebral body, it was retained as much as possible to reduce complications during the fixation and fusion process.

After removing the hardened wall reaction bone as much as possible, the bone bed was made conducive to accommodating the bone graft. The size of the bone graft bed was measured, and the three-sided cortical autologous iliac bone of the appropriate size was selected as the intervertebral support for bone grafting.

### Postoperative treatment and follow-up

After the operation, both groups of patients were observed for changes in vital signs. The serosanguineous drainage tube was removed after 48–72 h when the drainage volume dropped to less than 20–50 ml. In cases with large abscesses, the drainage tube was maintained for 8–10 days to ensure complete removal of residual bone, granulation tissue, or loose regenerated bone fragments. Generally, patients remained under strict bedrest for at least 3 weeks. During which time, the patients were encouraged to practice expectoration and short-term lower limbexercises to prevent further complications. After 3 weeks, the patients were able to be mobile with the protection of a brace.

After the operation, all patients were treated with anti-tuberculosis drugs for 2–7 months, and the drugs were adjusted or discontinued according to the patients’ medical profile. The liver and kidney functions were regularly examined during medication intake. Follow-up visits to the hospital at 1, 3, 6, 12, and 24 months after the operation were maintained to collect bloodwork, ESR, CRP, and X-rays of the reconstructed vertebrae. When necessary, complete CT and MRI examinations were conducted to review adjustments to the drugs. The symptoms of systemic tuberculosis were relieved by the end of the treatment.

### Evaluation index

Perioperative evaluation: The operation time, intraoperative blood loss, postoperative drainage volume, transfusion, and last VAS score were recorded in both groups.

Imaging evaluation: Cobb Angle measurement: An extension line was drawn on the upper endplate of the normal vertebral body above or adjacent to the diseased intervertebral space and the lower endplate of the next normal vertebra adjacent to the diseased vertebra. The angle between the two lines was the Cobb angle (defining the thoracolumbar and lumbar lordosis as positive, kyphosis as negative). Correction rate (CR) was defined as the following equation, CR = (preoperative kyphosis Cobb angle-postoperative kyphosis Cobb angle)/preoperative kyphosis Cobb Angle× 100%; loss Angle = kyphosis Cobb angle- postoperative kyphosis Cobb angle). According to the three-dimensional CT reconstruction, the evaluation of bone graft healing was as follows: (1) clarify that the bone trabecular connection formed a bone graft bridge throughout the bone graft area to; (2) visible fusion of the residual vertebrae to the bone graft; (3) marked disappearance of the bone graft interface.

Laboratory evaluation: ESR and CRP were measured before surgery, 6 months after surgery, and at the last follow up (62–66 months after surgery).

Postoperative neurological recovery: The Frankel Grade classification was used for the evaluation of the spinal cord recovery before surgery and at the last follow up (62–66 months after surgery).

Clinical efficacy: Clinical efficacy of both groups of patients was evaluated by the MacNab method [15] at the last follow up (62–66 months post surgery) and was divided into four grades: excellent, good, moderate, and poor. Excellent: no pain, unrestricted motor function, and the commencement of regular work and activity; Good: occasional pain, able to do light work; Moderate: some improvement, still feel pain, unable to work; Poor: nerve root damage, require further surgical treatment.

### Statistical processing

SPSS 21.0 statistical software was used for analysis. The measurement data was expressed as mean ± standard deviation **(**^**−**^***X ± S*)** and the counting data as a percentage (%). Multiple groups which means ESR, CRP and Cobb angle of preoperative and postoperative were compared by one-way analysis of variance. Age, course of disease, the operation time, intraoperative blood loss, postoperative drainage volume, transfusion were analysed by T-test and male/female, clinical efficacy and complications were used the chi-square (χ2) test or non-parametric test was used for the counting data. *P* < 0.05 was considered statistically significant.

## Results

### Perioperative evaluation indices

All patients had complete follow-up data. The patients in Group A and B followed up for 55–82 months and 50–86 months, respectively. The surgical time, intraoperative blood loss, postoperative drainage, and the need for blood transfusion in Group A significantiy better than Group B (*P* < 0.05). But there was no significant difference in the VAS score between the two groups at the last follow-up (55–82 months post surgery for Group A and 50–86 months post surgery for Group B) (*P* > .05) (Table 2).

**Table 2:** Comparison of the perioperative evaluation indices between the Groups A and B(^−^*X ± S*)

Observation index	Group A(*n* = 118)	Group B(*n* = 103)	Test value (*t/x*^*2*^)	*P*-value
Operation time (min)	219.45 ± 17.92	255.35 ± 29.79	*t* = 11.04	*P* = 0.000
Bleeding volume (ml)	714.92 ± 324.22	839.71 ± 355.49	*t* = 15.460	*P* = 0.000
Postoperative drainage (ml)	66.36 ± 17.78	97.09 ± 21.32	*t* = 11.68	*P* = 0.000
Blood transfusion (Yes/No)	18/100	28/75	*x*^*2*^ = 0.032	*P* = 0.022
Last follow-up VAS	0.86 ± 1.94	1.03 ± 0.96	*t* = 1.201	*P* = 0.231

### Imaging evaluation indices

There was no significant difference in the Cobb angle, Cobb angle correction rate and in the angle loss between Groups A and B before, after, and at the last follow-up (55–82 months post surgery for Group A and 50–86 months post surgery for Group B) (*P* > 0.05). This suggested that Group A was effective in correcting kyphosis caused by thoracolumbar and lumbar spinal tuberculosis, and was more conducive to the recovery of the physiology of the thoracolumbar and lumbar spine (Table 3).

**Table 3:** Comparison of the Cobb angle changes in Groups A and B before, after, and at the last follow-up (^−^*X ± S*)

Item	Cases	Preoperative(°)	Postoperative(°)	Last follow-up(°)	Loss(°)	Correction rate(%)
Group A	118	17.03 ± 18.95	27.80 ± 10.32	26.21 ± 8.77	1.61 ± 1.12	63.24 ± 8.26
Group B	103	15.91 ± 12.80	26.49 ± 7.05	25.39 ± 5.13	1.12 ± 1.06	66.50 ± 10.32
*t/χ*^2^	–	0.508	1.086	0.830	0.612	0.447
*P*-value	–	0.612	0.279	0.404	0.537	0.716

Spinal tuberculosis bone graft fusion was evaluated by CT three-dimensional reconstruction. The lesion cure rate was > 85% and > 95% at 6 months and 1 year after surgery. The bone graft was completely healed at the last follow-up (55–82 months postoperation for Group A and 50–86 months postoperation for Group B), and there was no statistical difference between Groups A and B. (*P* > 0.05) (Table 4).

**Table 4:** Comparison of bone graft healing between Groups A and B

Groups	Cases	6 months after surgery	1 year after surgery	Last follow-up
Group A	118	102(86.44%)	115(97.46%)	118(100%)
Group B	103	92(89.32%)	100(97.09)	103(100%)
*χ2*	–	0.425	0.06	–
*P*-value	–	0.514	0.806	–

### Laboratory test indicators

All patients tested positive for *Mycobacterium tuberculosis* before admission. There were no statistically significant differences in ESR and CRP between Groups A and B before surgery, 6 months after surgery, and at the last follow-up (55–82 months postoperation for Group A and 50–86 months postoperation for Group B) (*P*>, 0.05). ESR and CRP were close to normal at 6 months postoperation and were normal at the last follow-up (Table 5).

**Table 5:** Comparison of ESR and CRP before surgery, 6 months post surgery, and at the last follow-up in Groups A and B (^−^*X ± S*)

Groups	Cases	Preoperative		6 months after surgery		Last follow-up	
		ESR (mm/h)	CRP (mg/L)	ESR (mm/h)	CRP (mg/L)	ESR (mm/h)	CRP (mg/L)
Group A	118	37.49 ± 23.62	25.19 ± 22.17	14.69 ± 12.03	2.21 ± 1.11	8.37 ± 5.38	1.99 ± 0.89
Group B	103	37.40 ± 20.83	26.22 ± 23.13	12.53 ± 6.62	2.40 ± 1.34	7.60 ± 4.84	1.86 ± 0.69
*t*		0.31	−0.245	1.68	−1.16	1.113	1.21
*p*		0.976	0.807	0.094	0.248	0.267	0.225

Compared with group B, P > was 0.05, and the difference was not statistically significant

The normal range for ESR: male 0–15 mm/h, female 0–20 mm/h; the normal range for CRP 0–10 mg/l

### Postoperative neurological functions recovered

Neurological function at the last follow-up (55–82 months postoperation for Group A and 50–86 months postoperation for Group B) was significantly better than before surgery in both Groups A and B (Table 6).

**Table 6:** Frankel classification of neurological function at preoperative and final follow-up of Groups A and B

Groups	Cases	Grades	Preoperative	Last follow-up		
				C	D	E
A	118	B	5	0	2	3
		C	16		3	13
		D	27		3	24
		E	70			70
B	103	B	3	1	0	2
		C	10	0	2	8
		D	28		2	26
		E	62			62

### Clinical efficacy

The MacNab method was used to evaluate the clinical effectiveness of Groups A and B. The excellent and good rates of patients in both groups were 91.25 and 92.23% respectively, with no significant difference (*P*>, 0.05). The excellent and good rates of the last follow-up (55–82 months postoperation for Group A and 50–86 months postoperation for Group B) were 96.6 and 97.09% respectively, with no significant difference (*P* > 0.05)(Table 7).

**Table 7:** Comparison of the clinical efficacy in Groups A and B at 1 year post surgery and at the last follow-up

	Evaluation of clinical efficacy	excellent		good		moderate		poor	*U-value*
		Cases	%	Cases	%	Cases	%		
1 year after surgery	Group A	90	76.27	18	15.25	10	8.47	0	1.066
	Group B	85	82.52	10	9.71	8	7.77	0	
Last follow-up	Group A	102	86.44	12	10.16	4	3.39	0	0.641
	Group B	92	89.32	8	7.77	3	2.91	0	

Compared with group B, *P* > was 0.05, and the difference was not statistically significant

### Complications

Both groups had no severe neurological impairment, such as paraplegia, cauda equina syndrome, nerve root damage, cerebrospinal fluid leakage, and so on. The main complications are as follows. The complication rates in Groups A and B were 19.48% (23/118) and 23.30% (26/103) respectively, with no significant difference *χ*^*2*^ = 1.054, *P* = 0.305) (Table 8).

**Table 8:** Comparison of the postoperative complications in Groups A and B

Complications	Group A	Group B
Psoas abscess and	2	3
tuberculosis recurrence		
Incisional fat	4	2
liquefaction		
Incisional infection	3	2
Loose pedicle screws	2	2
& bone graft absorption		
Drug-related complication	12	10
Bone graft fractures	0	2
Vertebral degeneration	0	5

## Discussion

The surgical treatment has been shown to significantly increase cure rate, shorten course of treatment, reduce complications, and recurrence for spinal tuberculosis. Surgical treatment includes complete lesion removal, spinal canal decompression, deformity correction, bone graft fusion, internal fixation, and so on [16, 17].. The thoracolumbar and lumbar spines are the main sites of spinal tuberculosis. Therefore, in case of the thoracolumbar and lumbar tuberculosis, bone graft fusion and internal fixation are instrumental in the reconstruction of spinal stability [18, 19] and accelerated recovery of patients. However, the selection of bone graft fusion and internal fixation in the reconstruction of spinal tuberculosis has not generated much interest among scholars, and there is no unified standard. The method adopted by most scholars is to fix multiple standard motor units in addition to the fixation of the pathological motor unit [20, 21]. For thoracolumbar or lumbar spinal tuberculosis, the long-segment fixation is widely used. Gotzen [22] et al. first proposed the concept of a single-segment fixation, aka the diseased intervertebral fixation, under the principle of reducing fusion, fixing segments, and maintaining standard motor units. Since then, many doctors have found very good clinical results by performing basic [23–25] and clinical [12, 26, 27] studies on the treatment of spinal fractures with a single-segment fixation of the injured vertebrae. However, most authors still opt for a short- or long-segment fixation, in the surgical treatment of spinal tuberculosis, that is, non-disease intervertebral fixation.

The question still remains: is it possible to further standardize the method of spinal tuberculosis reconstruction surgery and shorten the scope of the operation reasonably and effectively? Is it possible to allow for a reliable spinal reconstruction that strengthens stability and minimizes fusion while repairing the diseased unit? Scholars at home and abroadhave conducted relevant biomechanical studies to determine whether the diseased intervertebral surgery can meet the requirements of spinal stability and load. Dick et al. [28] demonstrated enhanced spinal stability after a single-segment fixation in the injured vertebra. Similarly, our team successfully made a model of a defective bovine bone graft reconstruction using a single-segment fixation of the diseased vertebra. Both studies confirmed that a single segment fixation (i.e. the diseased intervertebral fixation) is sufficient to correct instability of the spine. These studies provide a strong theoretical basis for our clinical implementation of the diseased intervertebral fixation surgery.

Comparing the diseased intervertebral fixation with the non-diseased intervertebral fixation in thoracolumbar and lumbar tuberculosis, one finds that the latter produces more adjacent unit vicarious movement, increases concentrated stress on the adjacent segments, increases the intervertebral disc pressure, and ultimately, accelerates the degeneration of the adjacent segment thereby increasing the probability of ASD [29–31]. In this study, although there were no significant differences in the postoperative symptoms between the two groups, there were 5 cases of postoperative adjacent vertebral degeneration in the non-diseased intervertebral surgery group (Group B). Moreover, due to the fixation of additional motor segments in Group B, the operative time was prolonged and the intraoperative blood loss was increased. There was also a greater economic burden on the patients due to the additional fixations of non-diseased vertebrae. This study demonstrated that the diseased intervertebral surgery group (Group A) performed significantly better than the non-diseased intervertebral surgery group (Group B) in terms of operation time, intraoperative blood loss, postoperative drainage volume, and the requirement of blood transfusion during surgery.

The incidence of non-diseased intervertebral fixation pseudarthrosis has increased, and with that comes a higher risk of fractures and loosening of internal fixation after surgery; fragmented bones may move apart due to stress shielding making early fusion of the bone graft difficult; and the risk of bone graft absorption and displacement becomes elevated [32, 33]. In this study, There were no significant differences between the Cobb angle correction rate and the angle loss between the diseased and the non-diseased intervertebral surgery groups. Furthermore, the diseased intervertebral surgery was successful in correcting kyphosis caused by the thoracolumbar and lumbar spinal tuberculosis, and it is more conducive to the physiology of the thoracolumbar and lumbar spine. There was no significant difference in the recovery of curvature between the two groups of patients in terms of bone graft fusion rates. Based on these results, the diseased intervertebral surgery presents a more accurate and reasonable method for the surgical treatment of thoracolumbar and lumbar spinal tuberculosis. This operation does not sacrifice the adjacent normal exercise units and retains the spine’s motor function to the greatest extent. It is a relatively simple surgery, reduces the patient’s financial burden, and is shown to produce less fractures postoperation.

The internal fixation of the diseased vertebra alone obtains instant stability of the spine. However, the permanent stability of the reconstruction depends on the fusion of the spine [34]. In this study, all diseased intervertebral fixation patients underwent bone graft fusion in the diseased vertebra alone and no anterior or posterior normal motor unit fusions were allowed to occur, which is in stark contrast to the non-diseased intervertebral approach, which relies heavily on the degree of spinal fusion to determine the effectiveness of non-diseased intervertebral fixation. Meanwhile, whether the diseased intervertebral surgery can meet the requirements of the stability reconstruction of thoracic and lumbar tuberculosis, many scholars have carried out relevant biomechanical studies. Some scholars think, on the premise that the posterior column structure is complete, single-segment fixation not only meets the requirements of spine stability and load, but also the load of the nail-bar system of single-segment fixation is less than that of short-segment fixation, which is beneficial to reduce internal fixation-related complications [35]. Jin et al [36] demonstrated Single-segment pedicle screw fixation and correction surgery can fix and fuse the diseased segment in lumbar and sacral tuberculosis, retain normal movement in the adjacent spinal column, and promote functional recovery of the spinal column postoperatively. Bone grafting support is another essential component of the diseased intervertebral surgery. Studies with unsupported bone grafting revealed that the nail and rod stress of the fixation device increased significantly [37]. On the other hand, the intervertebral bone grafting support reduced the load and pressure of the posterior fixation device of the corresponding segment of the spine and produced a protective effect on the internal fixation device itself (i.e. protected from malformation and rod or screw breakage while promoting successful bone regeneration from the bone graft).

In China, our team first proposed the “complete lesion removal of spinal tuberculosis” by a large number of basic and clinical studies. Our previous research has shown that the sclerotic bone of affected vertebra plays an important role in blocking the antituberculosis drug’s penetration into tuberculosis focus [38]. Therefore, the sclerotic bone of affected vertebra was completely removed in our surgical operation, eliminating the barrier effect of the sclerotic bone, and effective drugs can fully kill *Mycobacterium tuberculosis*. We believe that after the complete lesion removal of spinal tuberculosis, pedicle screws were implanted into the subnormal bone tissue of the affected vertebra. *Mycobacterium tuberculosis* is less pathogenic than the pathogenic bacteria for purulent spondylitis, showing a low-viral infection and there have been reports in the literature that internal fixation with screws can be performed in the lesions of purulent infection of the spine, and the fixation is good [39].

Although the diseased intervertebral surgery shows promise in the treatment of thoracolumbar and lumbar tuberculosis, we must strictly grasp the indications and contraindications of its clinical application. Based on our study and literature review, the surgical indications include (1) non-rigid thoracolumbar and lumbar spinal tuberculosis; (2) the lesion is found in one spinal functional unit with no or ¼ pedicle damage; (3) intact upper and lower endplates of the diseased vertebrae; and (4) lesion vertebral Cobb less than 60°. The surgical contraindications include: (1) severe osteoporosis; (2) corneous kyphosis requiring correction of the thoracolumbar or lumbar tuberculosis; (3) posterior column thoracolumbar or lumbar tuberculosis; and (4) recurring tuberculosis with continuous multi-segment destruction. In addition. Principles of surgical selection for diseased intervertebral surgery is also crucial. According to our team’s long-term clinical research, we conclude that the following principles should be followed: 1) The lesion is relatively mild, only invading the intervertebral space and the vicinity of the adjacent endplate and located in one in the case of the side, choosing posterior surgery; 2) If the height of the remaining diseased vertebral body is greater than 2/3 of the normal vertebral body after the lesion is cleared, anterior approach is feasible, and the vertebral body nail is directly placed in the diseased vertebrae; 3) When the height of the remaining diseased vertebral body is 1/3 ~ 2/3 of the normal vertebral body height, the posterior and anterior approach can be selected, and the conventional pedicle screw-rod system is used for fixation; 4) When the remaining diseased vertebrae height is less than 1/3 of the normal vertebral body, the posterior and anterior approach can be selected. Short pedicle screws are inserted into the diseased vertebrae and fixed with a nail-rod system [37].

## Conclusion

Under the strict conditions of surgical indications, the diseased intervertebral surgery for the thoracolumbar and lumbar tuberculosis is safe, feasible, and can effectively restore physiological curvature of the spine while reducing degeneration of adjacent vertebrae; which is worthy of clinical application and promotion. Although the results of this study are satisfactory, there were some shortcomings. This study was a retrospective single-center case-controlled study with a low case study evidence level.

The incidence of fractures and fixation loosening after fixation of patients in the follow-up between the two groups was not explored. We will expand the sample size in further research to observe the abnormality of screws in the two groups of patients after surgery. Further studies are needed to explore the true benefit of the diseased intervertebral fixation surgery in the treatment of thoracolumbar and lumbar tuberculosis.

## Data Availability

The datasets used and/or analysed during the current study are available from the corresponding author on reasonable request.

## Abbreviations

VAS: Visual analog scale
CRP: C-reactive protein
ESR: Erythrocyte sedimentation rate
ASD: Adjacent segment disease
CT: Computed tomography
